# Supplementary material for: Differences in COVID-19 cyclicity and predictability among U.S. counties and states reflect the effectiveness of protective measures
Source: Sci Rep. 2023 Aug 31;13:14277. doi: 10.1038/s41598-023-40990-0 (PMC10471777; doi:10.1038/s41598-023-40990-0)
Supplement: Supplementary file 1 — Supplementary Information. [file 41598_2023_40990_MOESM1_ESM.pdf]

Supplementary Information for

Differences in COVID-19 cyclicity and predictability among U.S. counties and states reflect the effectiveness of protective measures

Claudio Bozzuto[1 *], Anthony R. Ives[2]

[1] Wildlife Analysis GmbH, Oetlisbergstrasse 38, 8053 Zurich, Switzerland. bozzuto@wildlifeanalysis.ch. ORCID 0000-0003-0355-8379

[2] Department of Integrative Biology, University of Wisconsin-Madison, Madison, WI 53706, USA. arives@wisc.edu. ORCID 0000-0001-9375-9523

[*] Corresponding Author: Claudio Bozzuto, Wildlife Analysis GmbH, Oetlisbergstrasse 38, 8053 Zurich, Switzerland. Phone: +41-44-550-40-30. bozzuto@wildlifeanalysis.ch

This PDF file contains:

- Supplementary Figures S1–S8
- Supplementary Table S1
- Additional methods information
- References Supplementary Information

Supplementary Figures

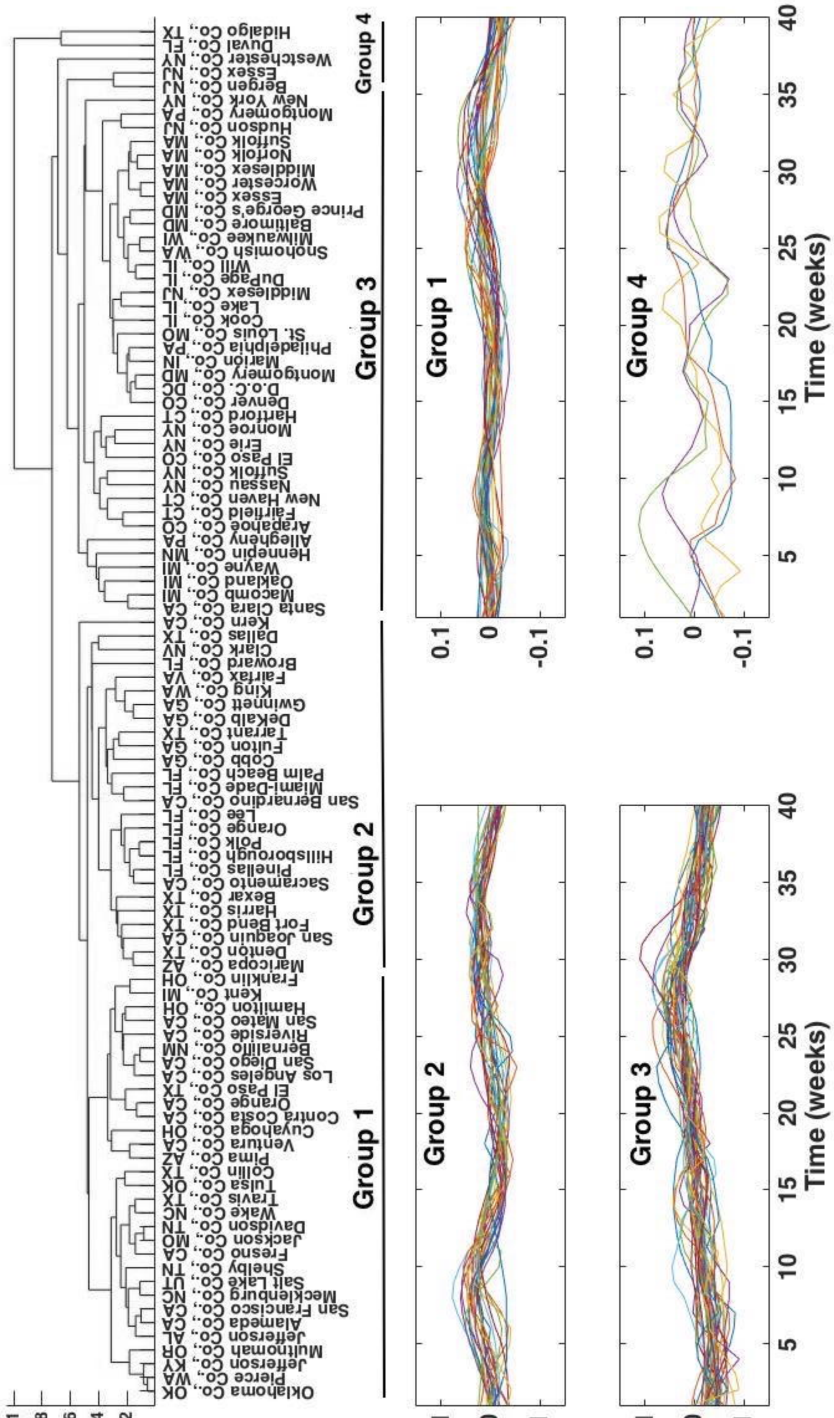

Fig. S1. Estimated *r(t)* time series at the county level. The estimated *r*(*t*) time series of the 100 analyzed counties are grouped according to the similarity of the dynamics. The similarity has been assessed using a dynamic time warping algorithm [1,2], and for illustrative purposes four groups have been used.

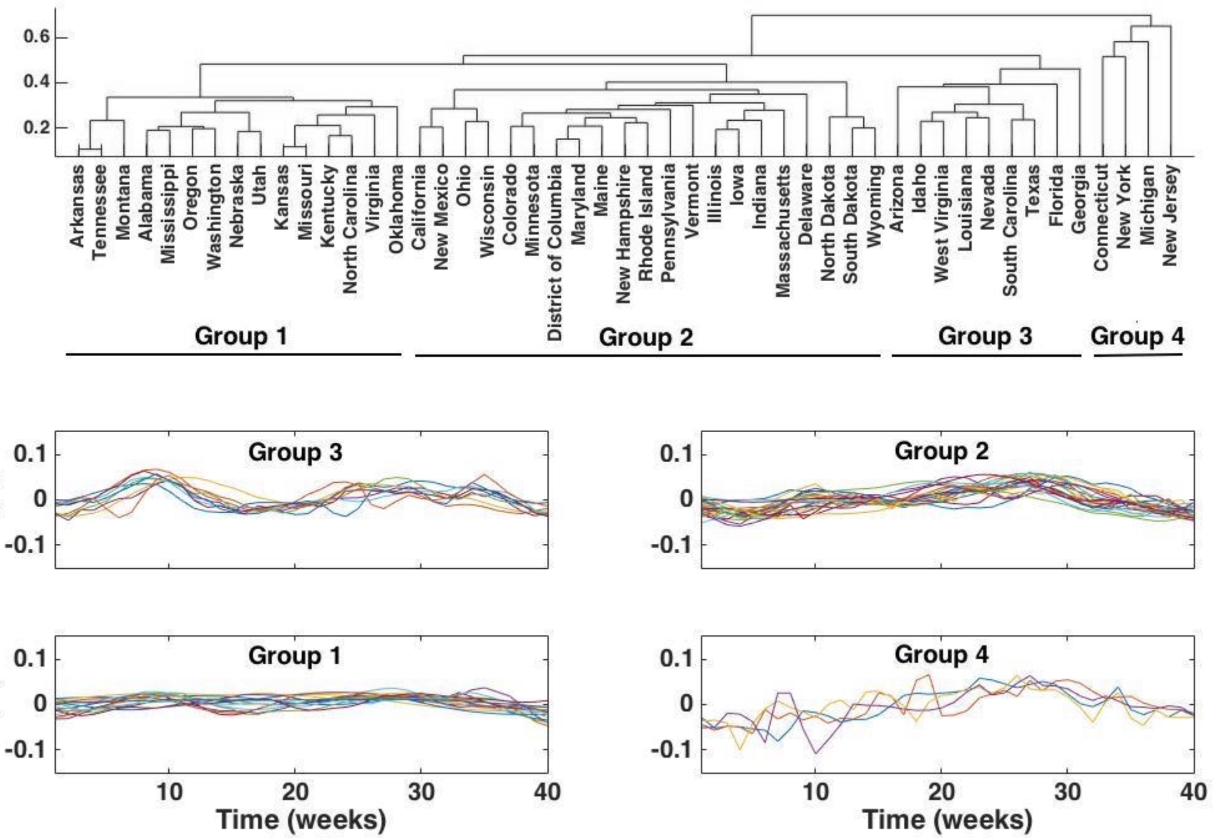

Fig. S2. Estimated *r*(*t*) time series at the state level. The estimated *r*(*t*) time series of the 49 analyzed states are grouped according to the similarity of the dynamics. The similarity has been assessed using a dynamic time warping algorithm [1,2], and for illustrative purposes four groups have been used.

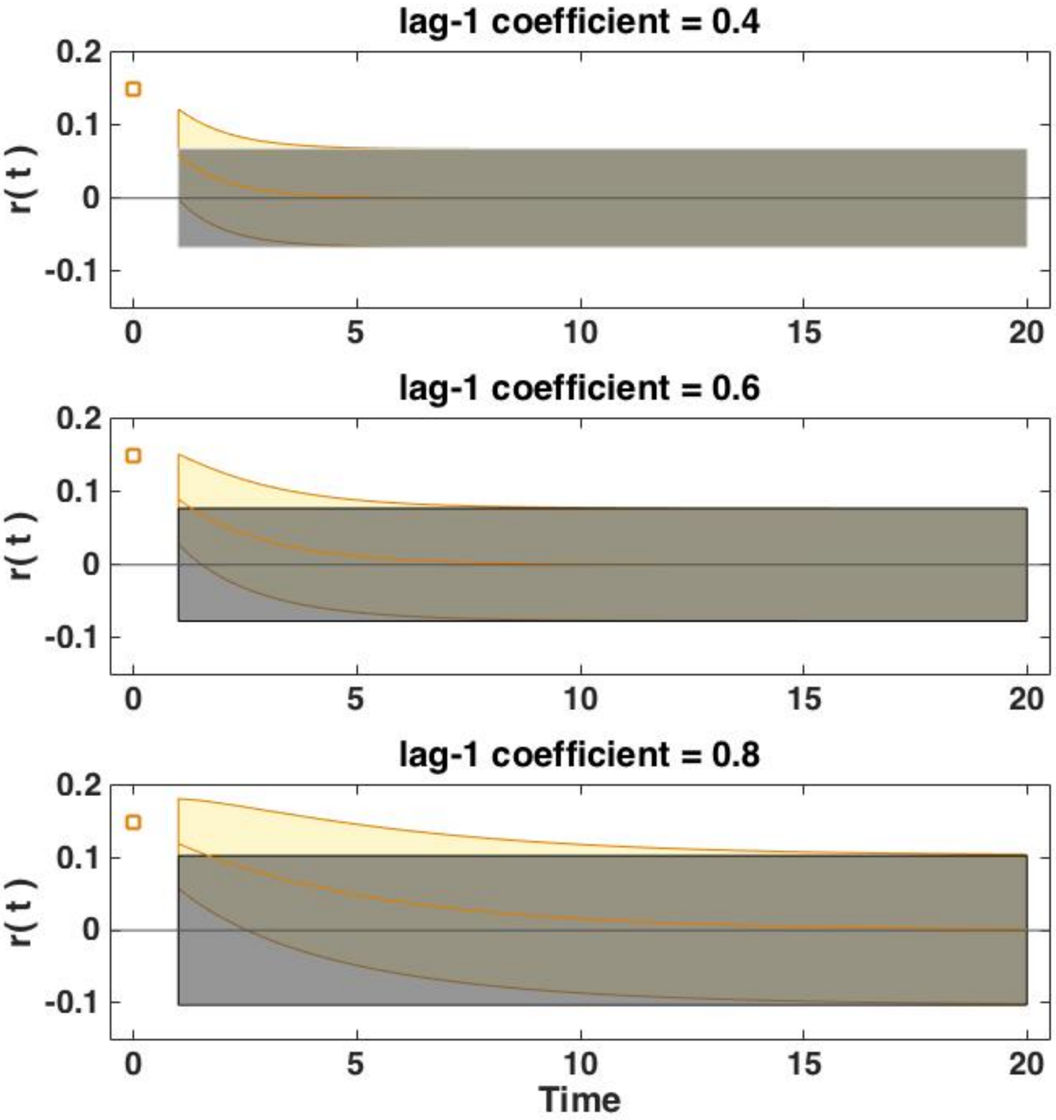

Fig. S3. Illustrative visualization of the transition distribution overlapping with the stationary distribution. For three illustrative AR(1) processes, the time-dependent transition distribution and the stationary distribution are shown, which are used to compute the predictability measure predictive power ($PP(t)$, eq. 2); the variances here are shown as 95% confidence intervals, and the orange square is an arbitrary initial state. The quicker the transition distribution is 'absorbed' by the stationary one, the faster predictability is said to be lost. Simulation parameters: lag-1 coefficients are given in the panel titles; $r(t = 0)$ = 0.15 and var(r) = 0.001 for all three AR(1) processes.

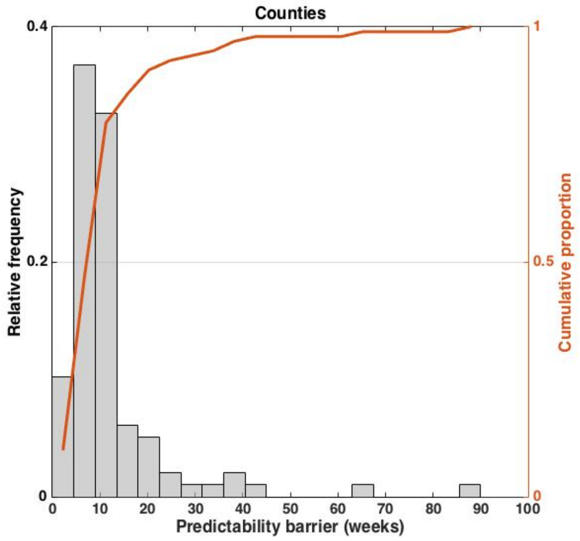

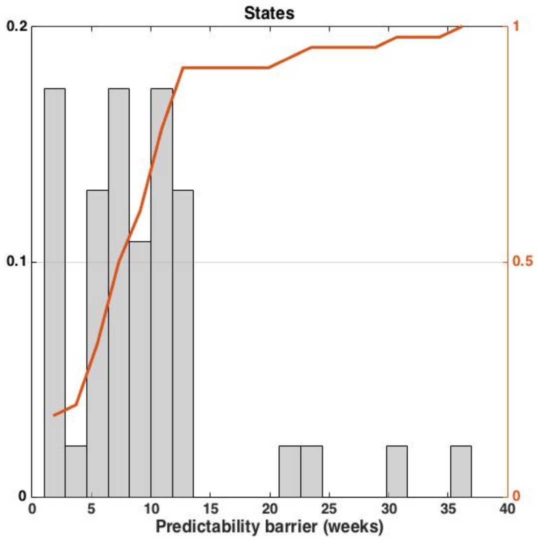

Fig. S4. Estimated predictability barrier. Shown are the distributions of the estimated predictability barriers (in weeks) at the county and state levels, along with the cumulative proportion of the respective jurisdictions.

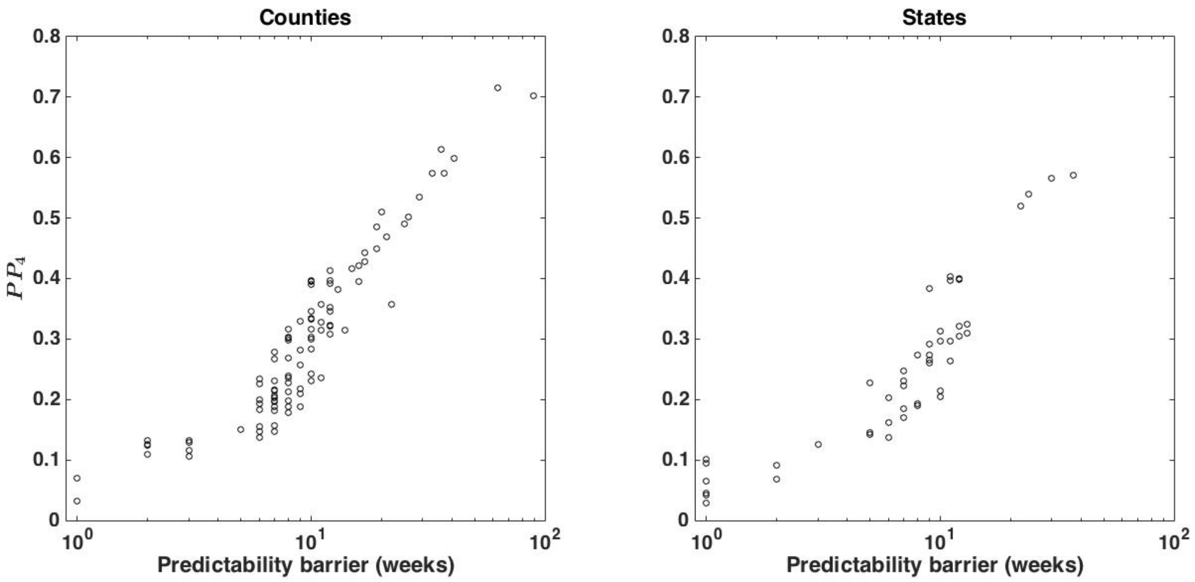

Fig. S5. ***PP_4* as a proxy for the predictability barrier**. The panels show that predictive power at time t = 4 (PP_4) is a good proxy for the predictability barrier (in weeks), at both the county and state levels.

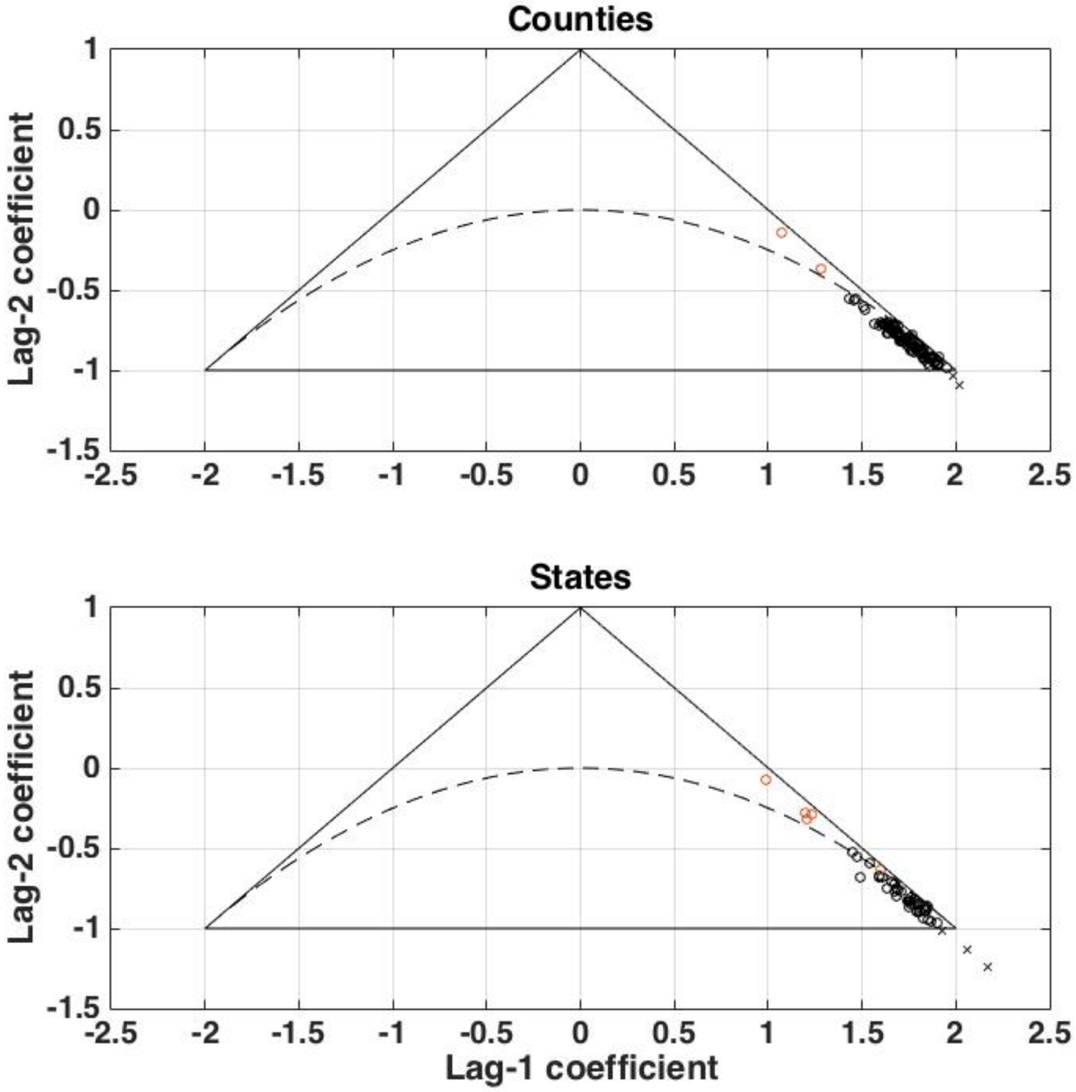

Fig. S6. Estimated autoregressive model parameters. Both panels show the estimated parameter pairs, i.e. the autoregressive lag-1 and lag-2 coefficients of the ARMA(2,2) model (eq. 1) fit to the $r(t)$ time series at the county level (cf. Fig. S1) and state level (cf. Fig. S2). Parameter combinations outside the triangle (marked with x's) imply a non-stationary process. Parameter combinations inside the triangle imply a stationary process, of which combinations below the parabola imply oscillatory dynamics (marked in black); estimated pairs implying non-oscillatory stationary dynamics are marked in red. The estimated lag-2 coefficients are approximately a linear function of the lag-1 coefficients: for counties, $b_2 \approx 0.80 - 0.93b_1$; for states, $b_2 \approx 0.87 - 0.97b_1$.

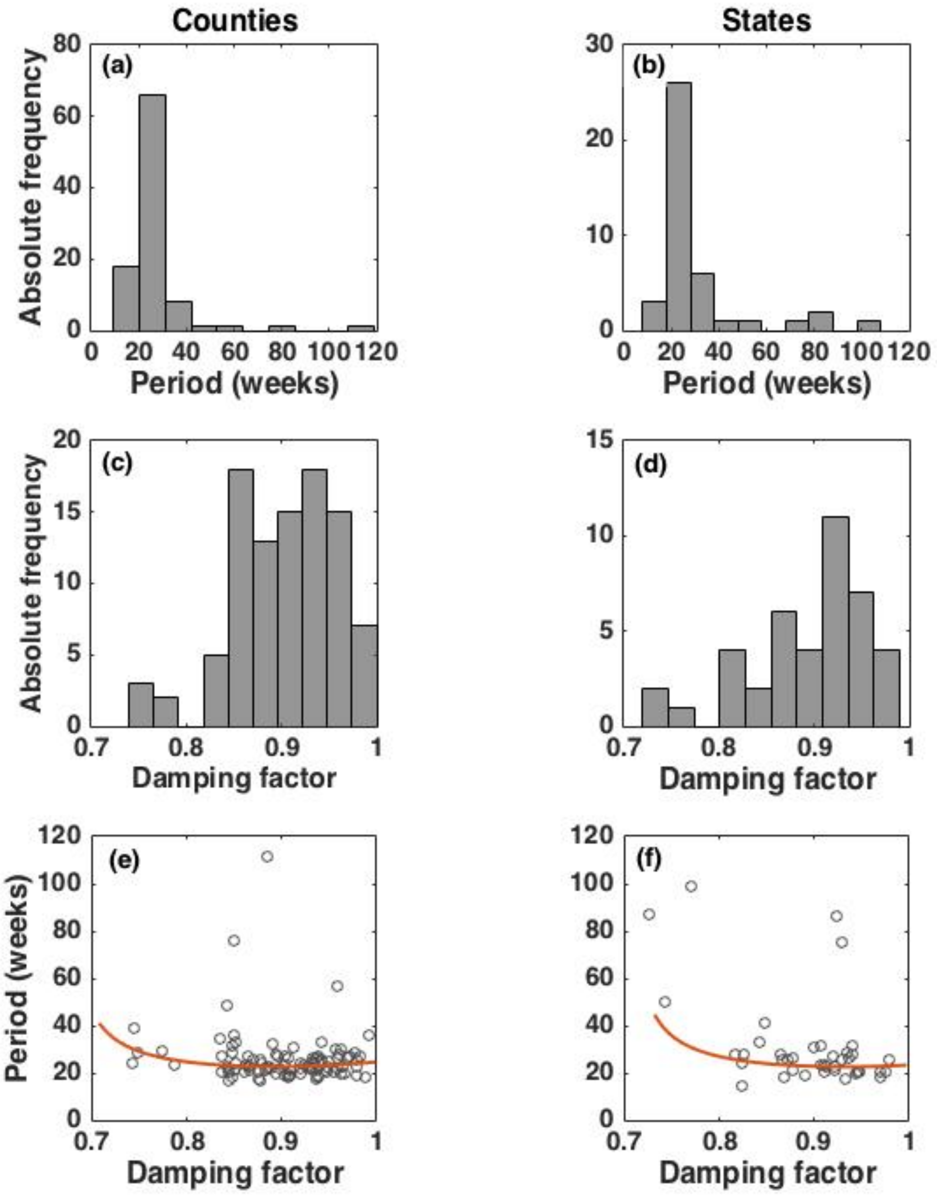

Fig. S7. Estimated average cycle length and damping factor. The two columns show, at the county and state level, **(a-b)** the distribution of the estimated average cycle length (period), **(c-d)** the distribution of the estimated damping factor, and **(e-f)** a scatter plot of the two measures. In the scatter plots, the red line was computed using the regression line for the two highly correlated AR(2) parameters b_1 and b_2 (cf. Fig. S4).

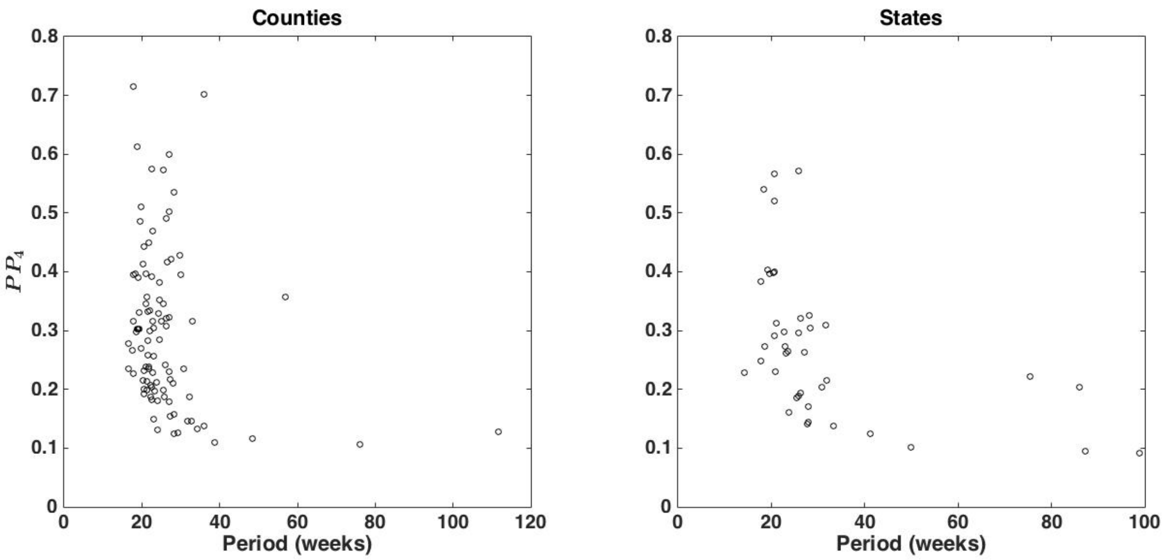

Fig. S8. Predictability versus period. The panels show, at the county and state levels, the estimated predictability as measured by predictive power at time $t = 4$ (PP_4) and the average cycle length (period), at both levels lacking a significant association.

Supplementary Tables

Table S1. Predictability barrier as a function of a preset threshold. This table presents a comparison of predictability barriers at the county and state levels, using two different preset thresholds, PP_{lim}: for one set of results PP_{lim} was computed using a threshold prediction R^2 of 0.25, corresponding to the results presented in the main text; for the second set of results, the threshold prediction R^2 was set to 0.10.

		**Threshold prediction R^2**	
		**0.25** (PP_{lim} = 0.1340)	**0.10** (PP_{lim} = 0.0513)
**Counties**	Predictability barrier: median (interquartile range)	9 weeks (7-12)	12 weeks (10-21)
	Predictability barrier scaled with respect to median cycle length (23 weeks)	40%	54%
	Proportion of counties with at least a fully predictable cycle length	10%	20%
**States**	Predictability barrier: median (interquartile range)	8 weeks (5-11)	12 weeks (9-17)
	Predictability barrier scaled with respect to median cycle length (26 weeks)	35%	50%
	Proportion of states with at least a fully predictable cycle length	5%	10%

Additional methods information

Estimation of COVID-19 spread rate *r*(*t*)

Here we give a summary description of our previously published approach[3] to estimate county-level and state-level spread rates *r*(*t*); please refer to the original publication for additional technical details. We employed a time-varying autoregressive model designed explicitly to reconstruct the non-observed time-varying spread rate using nonlinear, state-dependent error terms. The state-space model is

$$x(t) = r(t-1) + x(t-1),$$
$$r(t) = r(t-1) + \omega_r(t),$$
$$D(t) = \exp\big(x(t) + \phi(t)\big).$$

For every jurisdiction, the variable $x(t)$ is the unobserved, log-transformed value of weekly deaths at time t, and $D(t)$ is the observed count that depends on the observation uncertainty described by the random variable $\phi(t)$. The model assumes that the death count increases exponentially at rate $r(t)$, and this latent variable $r(t)$ changes through time as a random walk with $\omega_r(t) \sim \mathrm{N}(0, \sigma_r^2)$. We assume that the count data follow a quasi-Poisson distribution. Thus, the expectation of counts at time t is $\exp\big(x(t)\big)$, and the variance is proportional to this expectation.

We fit the model using the Kalman filter[4] to compute the maximum likelihood. In addition to the variances σ_r^2 and σ_ϕ^2, we estimated the initial value of $r(t)$ and the initial value of $x(t)$ at the start of the time series. Finally, we applied the Kalman smoother to generate time series of *r*(*t*) at the county and state levels. Note that the Kalman smoother is not an additional, 'external' method. Rather, after using the Kalman filter for parameter estimation and the reconstruction of r(t), the smoother seizes all information available after fitting and 'retrospectively adjusts' all time series involved in their entirety (e.g. 'true' death counts or unobserved spread rate); see Durbin and Koopman[4] for further details.

Predictive power for an ARMA(2,2) process

The variance of the transition distribution and the variance of the stationary distribution of the ARMA(2,2) model (eq. 1 in the main text) must be calculated to compute the predictability measure predictive power ($PP(t)$, eq. 2). This can be done by recasting the ARMA(2,2) model as a vector autoregressive process of order one, that is, a 4-dimensional VAR(1)[5]. To this end, the ARMA(2,2) model parameters populate the two jurisdiction-specific matrices

$$\mathbf{C} = \begin{bmatrix} b_1 & b_2 & a_0 & a_1 \\ 1 & 0 & 0 & 0 \\ 0 & 0 & 0 & 0 \\ 0 & 0 & 1 & 0 \end{bmatrix},$$

$$\mathbf{\Sigma} = \begin{bmatrix} \sigma^2 & 0 & \sigma^2 & 0 \\ 0 & 0 & 0 & 0 \\ \sigma^2 & 0 & \sigma^2 & 0 \\ 0 & 0 & 0 & 0 \end{bmatrix},$$

where $\mathbf{\Sigma}$ is a variance-covariance matrix, and σ^2 is the variance of the original univariate ARMA(2,2) process (eq. 1). The covariance matrix of the stationary distribution ($\mathbf{V}_\infty$) in vectorized form is defined as

$$\text{vec}\left(\mathbf{V}_{\infty(\text{VAR})}\right) \coloneqq (\mathbf{I} - \mathbf{C} \otimes \mathbf{C})^{-\mathbf{1}}\text{vec}(\mathbf{\Sigma}),$$

where $\text{vec}(\cdot)$ is the vec-operator, $\mathbf{I}$ is an identity matrix of appropriate size, $\otimes$ denotes the Kronecker product, and subscript VAR refers to the recast model. To compute the variance of the stationary distribution (as well as the transition distribution; see below) of the original univariate ARMA(2,2) process ($\mathbf{V}_{\infty(\text{ARMA})}$, which is a scalar), the two additional vectors

$$\mathbf{j} = [1 \quad 0 \quad 0 \quad 0],$$
$$\mathbf{h} = [1 \quad 0 \quad 1 \quad 0]$$

are needed, so that (superscript T means the transpose)

$$\mathbf{V}_{\infty(\text{ARMA})} = \mathbf{j}\ \mathbf{V}_{\infty(\text{VAR})}\ \mathbf{j}^{\text{T}}.$$

The time-dependent variance of the transition distribution of the original univariate ARMA(2,2) process ($\mathbf{V}_{\text{t(ARMA)}}$, which is a scalar) can be computed as

$$\mathbf{V}_{\mathrm{t(ARMA)}} = \sum_{i=0}^{t-1} \mathbf{\Phi}_i \, \sigma^2 \, \mathbf{\Phi}_i^{\mathrm{T}},$$

where the matrix $\mathbf{\Phi}_i \coloneqq \mathbf{j}\,\mathbf{C}^i\,\mathbf{h}^{\mathrm{T}}$; further technical details can be found in [5]. The transition distribution gives the evolution of the process through time, starting at a specific point r_0 at time t_0 (both potentially multi-dimensional) when the variance $\mathrm{var}(r_0) = 0$. We calculated the variance of the transition distribution for eq. 2 without considering spatial correlation, $\mathrm{cor}(\delta_i(t), \delta_j(t))$. The calculations are still correct for the marginal transition distribution, that is, the transition distribution for a single jurisdiction that does not depend on the other ones. Further, the differences in the mean spread rate among jurisdictions, $b_{0,i}$ (eq. 1), can also be ignored, because all estimates were nearly zero (*Results* in the main text).

Predictive power, estimation uncertainty, and structural uncertainty

In the preceding section we have shown how to compute predictive power, $PP(t)$ (eq. 2), for a univariate model as used in the present study. We have assumed that all parameters are known with certainty, which clearly is not realistic. The flip side of such approach, though, is that computation disregarding estimation uncertainty offers a 'best case predictability': explicitly considering estimation uncertainty will lower it[5,6]. In addition to estimation uncertainty, there is also structural uncertainty related to the model used[7,8]. In this section we offer some related general considerations along with references to specialized literature.

From eq. 2 in the main text it can be seen that in order to compute $PP(t)$ the variance of the transition distribution is scaled with respect to the variance of the stationary distribution. This ratio, $\mathbf{V}(t)\mathbf{V}_\infty{}^{-1}$, can be expanded to include estimation uncertainty. Both the transition and the stationary variance can be expressed as sums[5,6],

$$\mathbf{V}(t) = \mathbf{V}'(t) + \mathbf{E}(t),$$
$$\mathbf{V}_\infty = \mathbf{V}'_\infty + \mathbf{E}_\infty,$$

where we use a prime to designate an exactly known variance, and the second summand in each equation is the covariance matrix stemming from estimation; if we set $\mathbf{E}(t) = \mathbf{E}_\infty \equiv 0$, we recover $PP(t)$ as computed in the previous section. The computation of $\mathbf{E}(t)$ and $\mathbf{E}_\infty$ is detailed in Lüktepohl[5] (ch. 3), including how to iteratively compute $\mathbf{E}(t)$ for any forecast

horizon t. Nonetheless, from a practical perspective the computation of $PP(t)$ under estimation uncertainty is probably easiest using a parametric bootstrap using the estimated uncertainty around the parameters needed to compute $PP(t)$. The main insight here is that including estimation uncertainty will reduce predictive power[6], and therefore using parameter point estimates to compute $PP(t)$ can be regarded as indicating a 'best case predictability': if the latter is high, then including estimation uncertainty might be a cautious approach for public health decision-making.

Finally, there is also the issue of uncertainty related to a model used, i.e. structural uncertainty[7,8]. An often-employed way to address model uncertainty is to select a 'best' model among competing models using an information criterion like AIC (or the small-sample version AICc) and subsequently assume the selected model is the correct or 'true' one. A general problem arises from using the same data – quite understandably for short time series – for model selection and parameter estimation, often resulting in overly confident predictions, i.e. narrow prediction intervals (Ref.[7,8] and references therein). More fundamentally, there is no simple solution to tackle structural uncertainty[8]. One approach could be to concomitantly use several sensible models using model averaging (e.g. Ref.[9,10]). For example, Cramer *et al.*[11] have shown the usefulness of using ensemble forecasts during the COVID-19 pandemic. Nonetheless, in our study we refrain from applying such approach as it is not clear to us how to interpret averaged predictive power values (see also caveats expressed in Ref.[12]).

Simulation model

We designed the simulation model to determine the plausibility of the hypothesis that changes in the transmission rate of COVID-19 in response to death counts could explain patterns of cyclicity and predictability observed in state and county data. The simulation model is a modification of that presented in Ref.[3]. The simulation model tracks the epidemic on a daily time scale and explicitly includes the time period from infection to subsequent transmission (infectiousness), and from infection to death; therefore, it is akin to a SEIR model.

The simulation model tracks the number of infected individuals on day t who were infected τ days previously, $I(t;\tau)$. After 25 days, they are all assumed to be recovered or dead. The relative daily infectiousness of an infected individual, $p(t)$, is given by a Weibull distribution with mean 7.5 days and standard deviation 3.4[6] (Supplementary Fig. 3a in Ref.[3]). For an individual who dies, the day of death, $d(t)$, is given by a Weibull distribution with mean 18.5 days and standard deviation 3.4[6] (Supplementary Fig. 3b in Ref.[3]). Finally, the

time between initial infection and diagnosis, $h(t)$, is assumed to be log-normally distributed with mean 5.5 days and standard deviation 2.2[8] (Supplementary Fig. 3c in Ref.[3]).

Deaths occur according to the infection age category of individuals, $I(t;\tau)$, so that the probability of death of individuals that had been infected τ days earlier is $(1-s)d(\tau)$. Here, s is the overall survival probability, assumed to be s = 98%; changes in this assumption had little effect on the simulation results. Once an individual dies, they are removed from the pool of individuals. These individuals are summed to calculate the daily death count, $D(t)$.

On day t, the number of new infections is $\beta(t)S(t)\exp(\alpha(t))\sum_\tau p(\tau)I(t;\tau)$, where $\beta(t)$ is the transmission rate and $p(\tau)$ scales the transmission rate by the infectiousness of individuals infected τ days earlier, $I(t;\tau)$. Here, we let $\beta(t)=\beta_{\text{const}}\left(1+\omega D(t-14)\right)^{-1}$, so that an increasing number of deaths two weeks beforehand decreases the transmission rate below the constitutive transmission rate of β_{const}; this transmission rate function is the same as in eq. 3 in the main text, albeit here expressed on a daily timescale. The lognormal random variable $\exp(\alpha(t))$ is included to represent environmental variation; its variance was selected to mimic the variation in $r(t)$ observed in the real data. Note that the simulations produce values of $r(t)$ that we analyzed directly, rather than estimating $r(t)$ by applying a Kalman smoother[4] to the simulated values of $D(t)$.

Although the time series were generated on a daily time scale, simulated data were subsequently aggregated to weeks for analyses. The analyses of $r(t)$ were performed on time series of length 40 weeks. The results of the analyses depended on the initial number of infected individuals and the week at which the analyses were initiated. For the analyses in the main text, we initially simulated 40 weeks of data starting with $I(t;\tau)$ = 10^{-6}. Other parameters were $\omega = 10^5$ and the variance of $\alpha(t)$ = 0.03.

We analyzed each simulated time series separately, unlike the real time series which were all analyzed simultaneously. Furthermore, fits using an ARMA(2,2) were unstable for time series of length 40, and therefore we fit the simulated time series using an AR(2) model, estimating PP_4, the damping factor d, and average cycle length (period) from the autoregressive coefficients in the same way as the real data.

References Supporting Information

1. Müller, M. Dynamic Time Warping. in *Information Retrieval for Music and Motion* 69–84 (Springer, 2007).
2. Wang, Q. *Dynamic Time Warping (DTW)*. https://github.com/wq2012/dynamic_time_warping/releases/tag/v2.2 (2023).
3. Ives, A. R. & Bozzuto, C. Estimating and explaining the spread of COVID-19 at the county level in the USA. *Communications Biology* **4**, 1–9 (2021).
4. Durbin, T. late J. & Koopman, S. J. *Time Series Analysis by State Space Methods*. (Oxford University Press, 2012).
5. Lütkepohl, H. *New Introduction to Multiple Time Series Analysis*. (Springer, 2005).
6. Schneider, T. & Griffies, S. M. A Conceptual Framework for Predictability Studies. *Journal of Climate* **12**, 3133–3155 (1999).
7. Draper, D. Assessment and Propagation of Model Uncertainty. *Journal of the Royal Statistical Society: Series B (Methodological)* **57**, 45–70 (1995).
8. Chatfield, C. & Xing, H. *The Analysis of Time Series: An Introduction with R*. (CRC Press, Taylor & Francis Group, 2019).
9. Dormann, C. F. *et al.* Model averaging in ecology: a review of Bayesian, information-theoretic, and tactical approaches for predictive inference. *Ecological Monographs* **88**, 485–504 (2018).
10. Burnham, K. P. & Anderson, D. R. *Model Selection and Multimodel Inference: A Practical Information-Theoretic Approach*. (Springer Science & Business Media, 2003).
11. Cramer, E. Y. *et al.* Evaluation of individual and ensemble probabilistic forecasts of COVID-19 mortality in the United States. *Proceedings of the National Academy of Sciences* **119**, e2113561119 (2022).
12. Pennekamp, F. *et al.* The intrinsic predictability of ecological time series and its potential to guide forecasting. *Ecological Monographs* **89**, e01359 (2019).
